# Reversible complete atrioventricular block in immune checkpoint inhibitor–associated triple-M overlap syndrome: a case report

**DOI:** 10.1093/ehjcr/ytag595

**Published:** 2026-08-08

**Authors:** Sonal Prasad, Michael Dicaro, Thomas Iida, Tina Frisch, Mutsumi Kioka

**Affiliations:** Division of Pulmonary and Critical Care Medicine, Department of Internal Medicine, Kirk Kerkorian School of Medicine, University of Nevada, Las Vegas, NV 89102, USA; Division of Cardiovascular Medicine, Department of Internal Medicine, Kirk Kerkorian School of Medicine, University of Nevada, Las Vegas, NV 89102, USA; Department of Emergency Medicine, Kirk Kerkorian School of Medicine, University of Nevada, Las Vegas, NV 89106, USA; Department of Pharmacy University Medical Center, Las Vegas, NV 89102, USA; Division of Pulmonary and Critical Care Medicine, Department of Internal Medicine, Kirk Kerkorian School of Medicine, University of Nevada, Las Vegas, NV 89102, USA

**Keywords:** Immune checkpoint inhibitors, Triple-M overlap syndrome, Myocarditis, Complete atrioventricular block, Ovarian cancer, Case report

## Abstract

**Background:**

Immune checkpoint inhibitors (ICIs) are increasingly used in the treatment of advanced malignancies but are associated with rare and potentially life-threatening immune-related adverse events. Overlap syndromes involving myocarditis, myositis, and myasthenia gravis-referred to as Triple-M overlap syndrome-have been reported; however, reversible high-grade atrioventricular block remains uncommon.

**Case summary:**

A 74-year-old woman with advanced ovarian cancer treated with pembrolizumab presented with dyspnoea, myalgia, and right-sided ptosis 22 days after ICI therapy. Electrocardiography demonstrated ST-segment elevation in the anterior leads with markedly elevated high-sensitivity troponin. On the following day, persistent ST-segment elevation prompted evaluation for acute myocardial infarction, and coronary angiography revealed no obstructive coronary artery disease. The clinical course was complicated by sustained ventricular tachycardia followed by complete atrioventricular block requiring temporary transvenous pacing. Laboratory evaluation showed markedly elevated creatine kinase, and neurological findings were consistent with neuromuscular junction involvement. Immune-mediated Triple-M overlap syndrome was suspected, and high-dose corticosteroids and intravenous immunoglobulin were initiated. The patient demonstrated progressive clinical improvement, with resolution of arrhythmias and recovery of atrioventricular conduction.

**Discussion:**

This case highlights a rare presentation of ICI-associated Triple-M overlap syndrome complicated by reversible complete atrioventricular block. Immune-mediated conduction abnormalities may progress despite improving cardiac biomarkers, and early immunomodulatory therapy may enable reversibility of high-grade atrioventricular block.

Learning points

- ICI-associated myocarditis may mimic STEMI despite normal coronary arteries.
- High-grade AV block in Triple-M overlap syndrome may be reversible with early immunomodulatory therapy

## Background

Immune checkpoint inhibitors (ICIs) have become an integral component of modern cancer therapy and have markedly improved outcomes across a wide range of malignancies. Monoclonal antibodies targeting the programmed cell death-1 (PD-1) receptor, and its ligand PD-L1, have received approval for multiple oncologic indications.^1^ Despite their significant therapeutic efficacy, ICIs are associated with a diverse range of immune-related adverse events (irAEs) that can affect multiple organ systems, most notably the cardiovascular, neurologic, and musculoskeletal systems.^2^

## Summary figure

**Figure:**
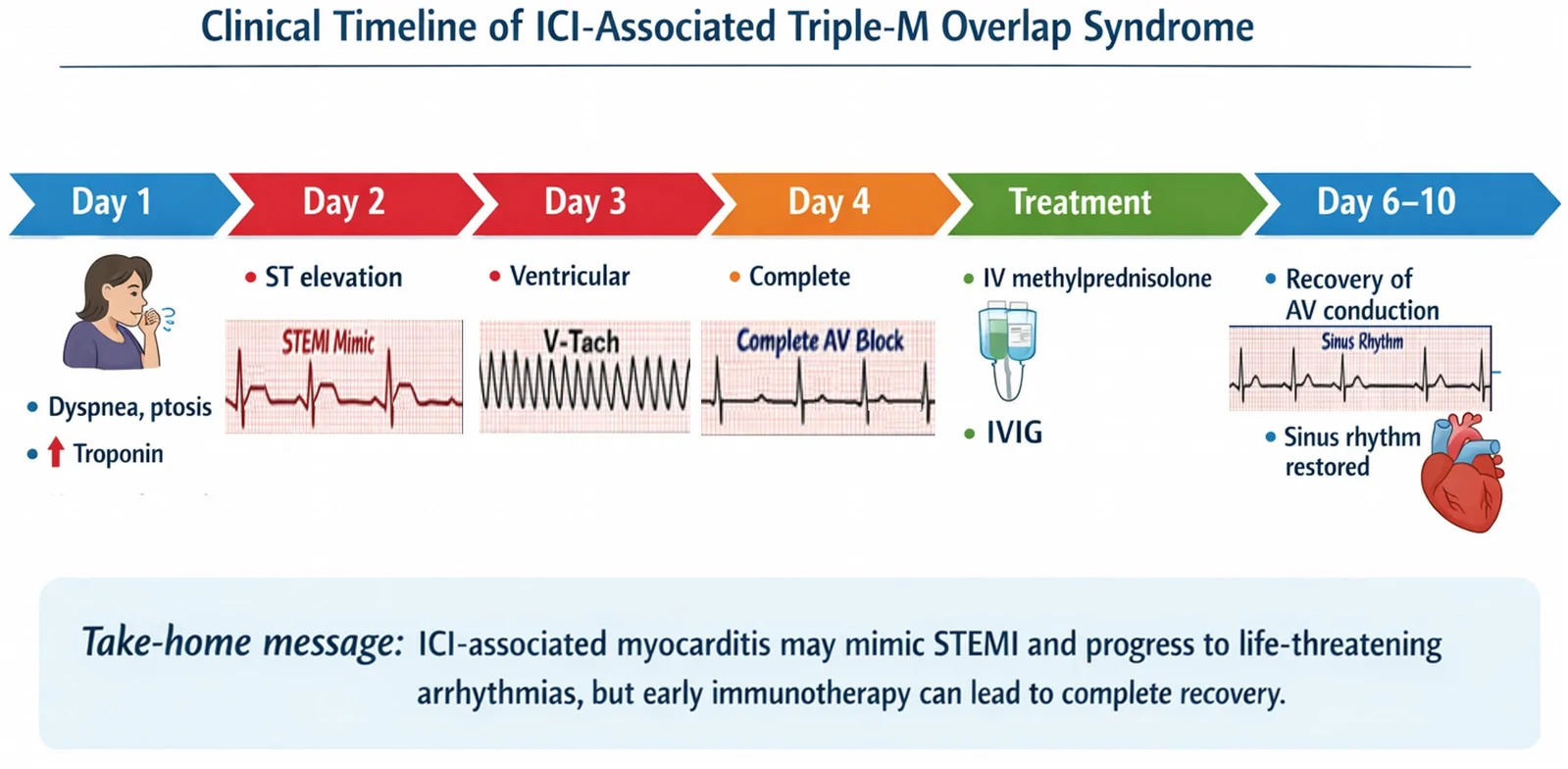


We present a case of Triple-M overlap syndrome associated with ICI therapy, characterized by myocarditis, myositis, and myasthenia gravis, and complicated by a reversible complete atrioventricular (AV) block.^3^ This case highlights a rare but potentially life-threatening manifestation of immune-related cardiotoxicity and underscores the importance of early recognition of overlapping immune-mediated syndromes.

## Case presentation

A 74-year-old woman with advanced ovarian cancer (histologic subtype unavailable), hypertension, and hyperlipidaemia, status post hysterectomy and bilateral oophorectomy, with a history of ureteral stent placement for a surgical complication 6 years prior to presentation, was receiving pembrolizumab and bevacizumab in the outpatient setting.

She presented to the emergency department 22 days after her most recent ICI infusion with shortness of breath, back and thigh pain, and right-sided ptosis that had been present for several days. On presentation, the patient appeared uncomfortable. Physical examination was notable for right-sided ptosis and bilateral thigh tenderness to deep palpation without swelling or deformity. The remainder of the cardiopulmonary and neurological examination was unremarkable. Vital signs demonstrated a blood pressure of 86/62 mmHg, heart rate of 128 bpm, respiratory rate of 31/min, and oxygen saturation of 95% on room air. Laboratory findings included a potassium level of 4.0 mmol/L (3.5–5.1 mmol/L), magnesium 1.57 mg/dL (1.60–2.60 mg/dL), and calcium 10.0 mg/dL (8.4–10.2 mg/dL). Venous blood gas analysis showed a pH of 7.48 (7.35–7.45) and a pCO_2_ of 27.0 mmHg (35.0–45.0 mmHg). Electrocardiography (ECG) demonstrated sinus tachycardia, and high-sensitivity troponin I was markedly elevated at 22 380 ng/L (≤34 ng/L). Although the troponin level was markedly elevated, the patient’s presenting symptoms and initial electrocardiographic findings were not considered sufficiently suggestive of an acute coronary syndrome to warrant emergent coronary angiography. The patient was therefore managed with close clinical observation and serial ECG monitoring. A baseline ECG was unavailable because the patient was visiting from out of town. During admission, a transthoracic echocardiogram showed a normal-sized left ventricle with mild concentric hypertrophy, and the estimated ejection fraction was greater than 55%. The right ventricle was normal in size and function. There were no regional wall motion abnormalities or pericardial effusion. (see Supplementary material online, *Files S1–S4* for representative echocardiographic video clips and the complete echocardiography report). Cardiac magnetic resonance imaging (MRI) was not performed due to limited availability at our institution.

**Figure 1:**
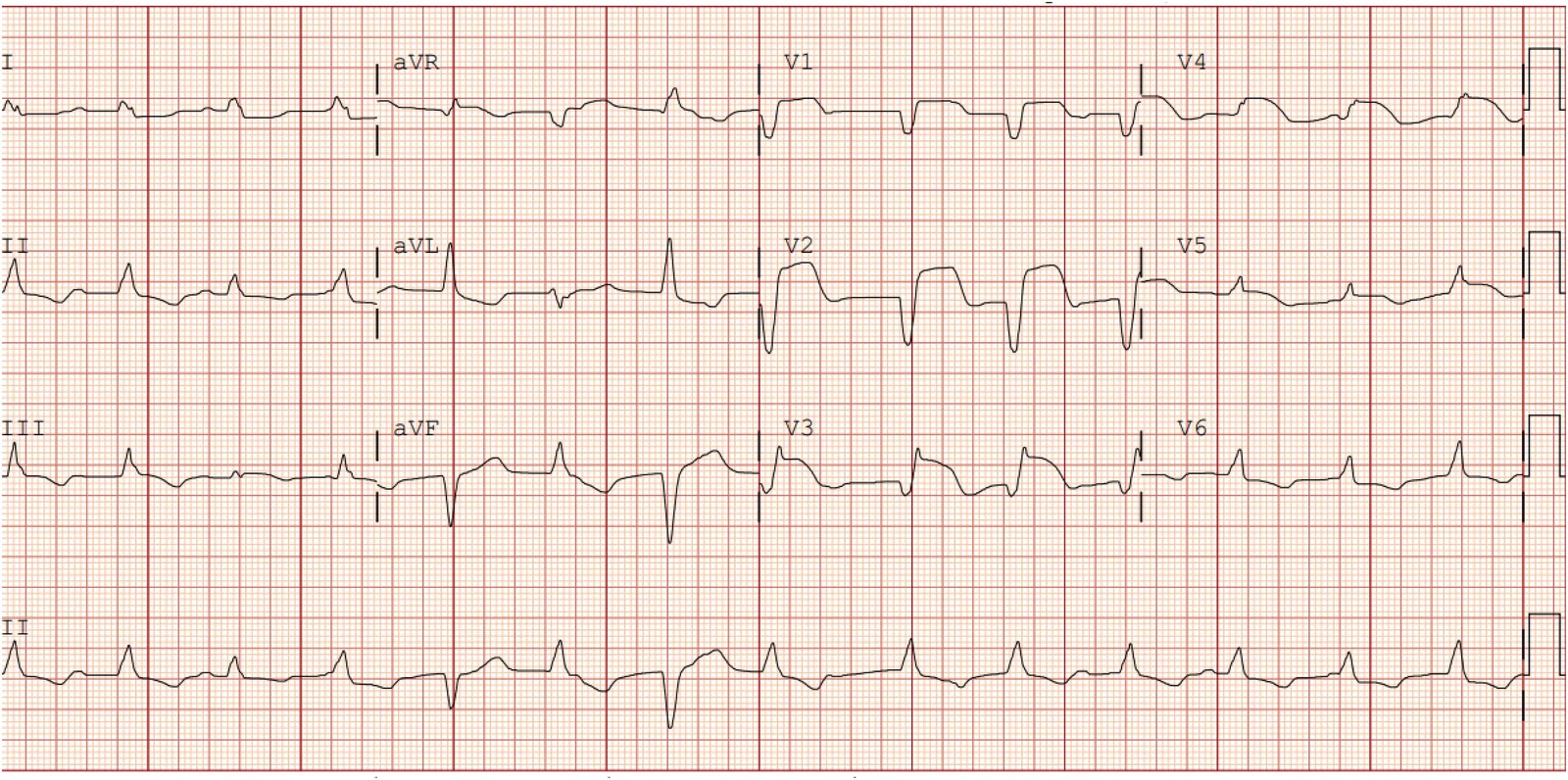
Initial 12-lead electrocardiography showing ST-segment elevation in the anterior precordial leads (V1–V4). Retrospective review suggested early conduction system involvement with atrioventricular (AV) dissociation and ventricular ectopy preceding clinically recognized complete AV block.

The next day, ECG demonstrated ST-segment elevation and markedly elevated troponin levels, raising concern for acute ST-segment elevation myocardial infarction (*Figure 1*). Coronary angiography was subsequently performed and revealed no obstructive coronary artery disease (see Supplementary material online, *File S5*). Retrospective review of this ECG also revealed findings suggestive of early conduction system involvement, including AV dissociation, left bundle branch block morphology, lack of the expected tachycardic response despite anterior ST-segment elevation, and ventricular ectopy, raising the possibility that complete AV block was already developing at this stage. Laboratory evaluation showed a significantly elevated creatine kinase level of 8394 U/L (30–200 U/L), correlating with the patient’s complaints of thigh and back pain. Neurologic examination was notable for right-sided ptosis, which demonstrated transient improvement with an ice pack test, raising concern for neuromuscular junction involvement (see Supplementary material online, *File S6*).

**Figure 2:**
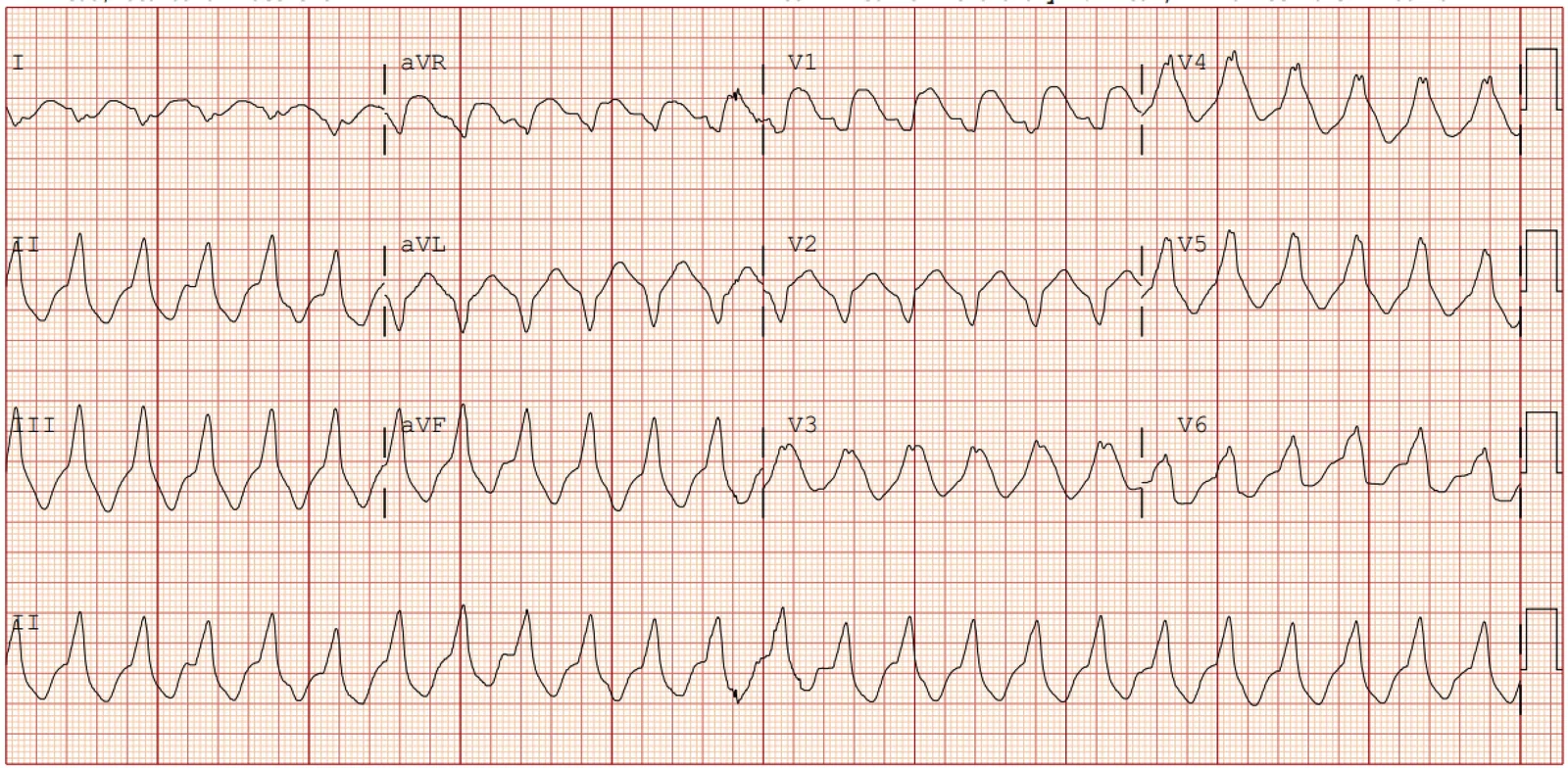
12-Lead electrocardiography demonstrating wide-complex tachycardia consistent with ventricular tachycardia at a rate of approximately 142 bpm.

**Figure 3:**
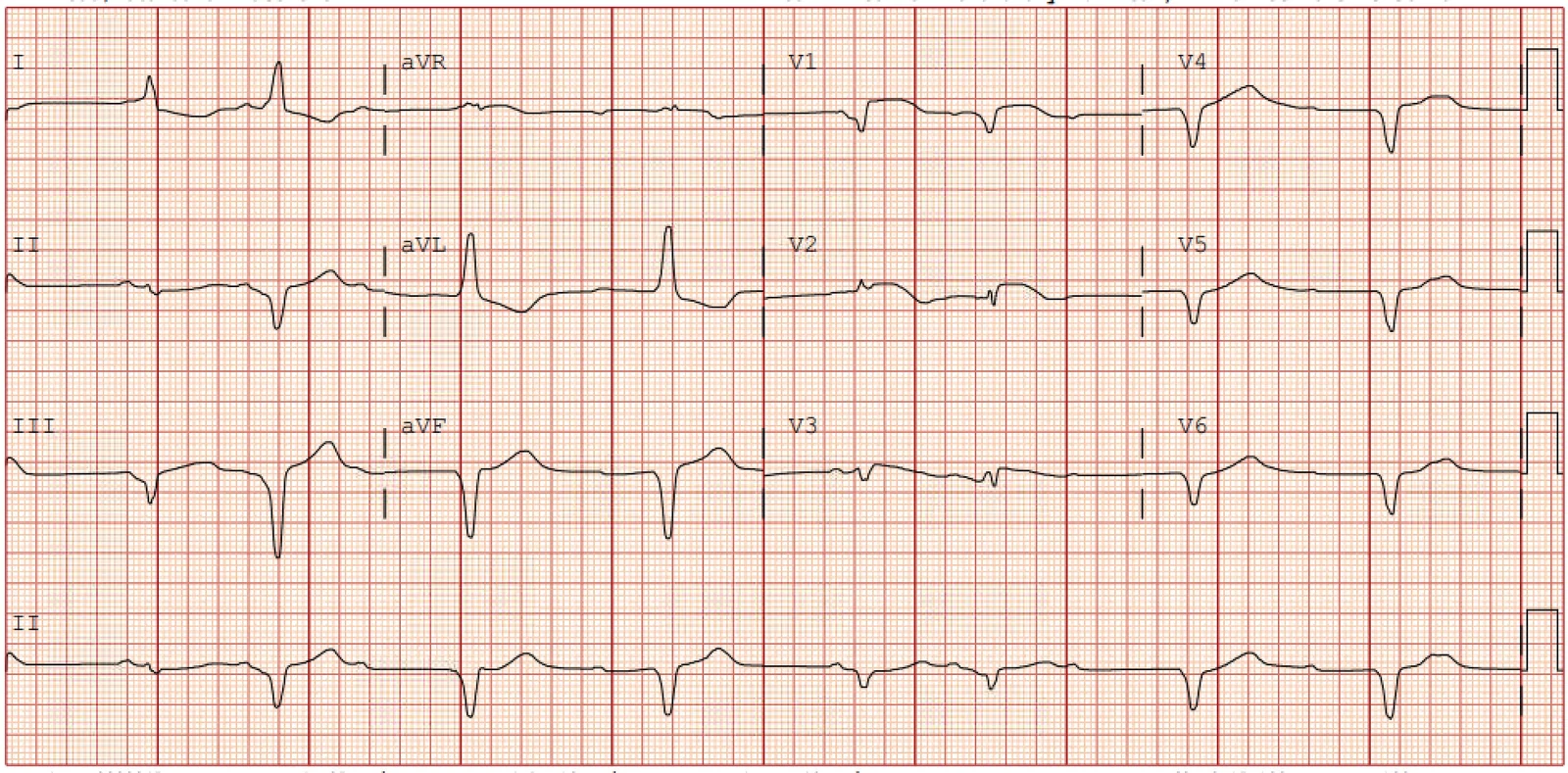
Electrocardiography demonstrating complete atrioventricular (AV) block with atrioventricular dissociation after treatment for ventricular tachycardia.

**Figure 4:**
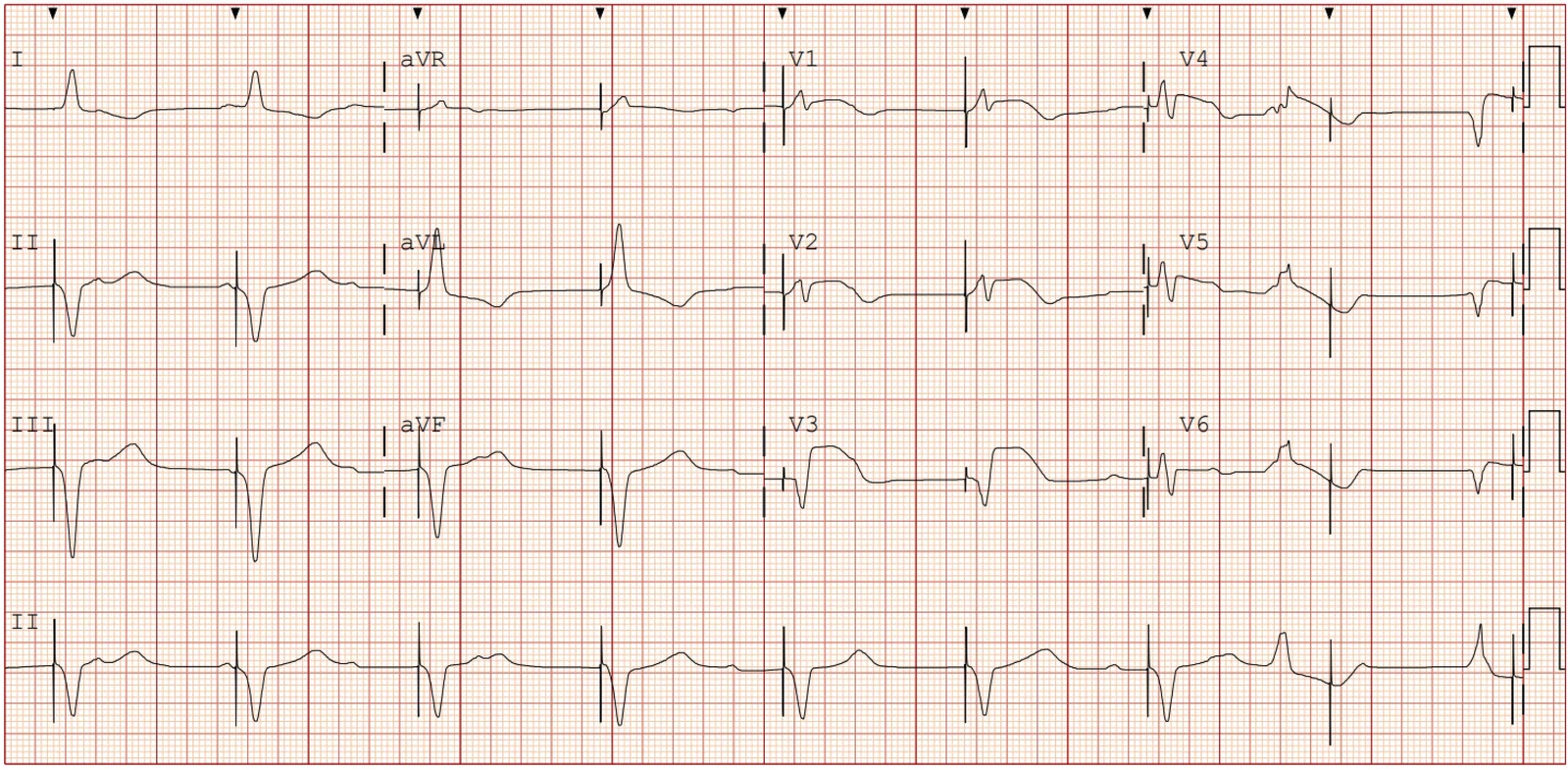
Temporary transvenous pacing. Electrocardiography obtained after emergent placement of a temporary transvenous pacemaker, demonstrating paced ventricular rhythm.

On Hospital Day 3, the patient developed sustained ventricular tachycardia at approximately 141 bpm (*Figure 2*). At the time of the arrhythmia, she remained alert, denied chest pain, and had a blood pressure of 93/56 mmHg. She was treated with an amiodarone infusion; however, due to refractory ventricular arrhythmia, therapy was transitioned to lidocaine. Although the ventricular tachycardia resolved, ECG on Hospital Day 4 revealed complete AV block (*Figure 3*), necessitating placement of a temporary transvenous pacemaker (*Figure 4*).

Given the constellation of myocarditis, myositis, and neuromuscular findings in the setting of recent ICI therapy, an immune-mediated adverse event was suspected. Although ICI-associated Triple-M overlap syndrome was considered early in the clinical course, the diagnosis required multidisciplinary evaluation and exclusion of alternative aetiologies before a consensus diagnosis was reached. High-dose immunomodulatory therapy was initiated on Hospital Day 3 with intravenous immunoglobulin at 0.4 g/kg/day and intravenous methylprednisolone 1 g daily (treatment course summarized in Supplementary material online, *File S7*). Neurology was consulted for further evaluation. Serologic testing revealed negative acetylcholine receptor–binding, blocking, and modulating antibodies, as well as a negative antinuclear antibody. Smooth muscle antibody was mildly elevated at 37 units (reference >30, moderate positive) with a speckled pattern greater than 1:1280.

**Figure 5:**
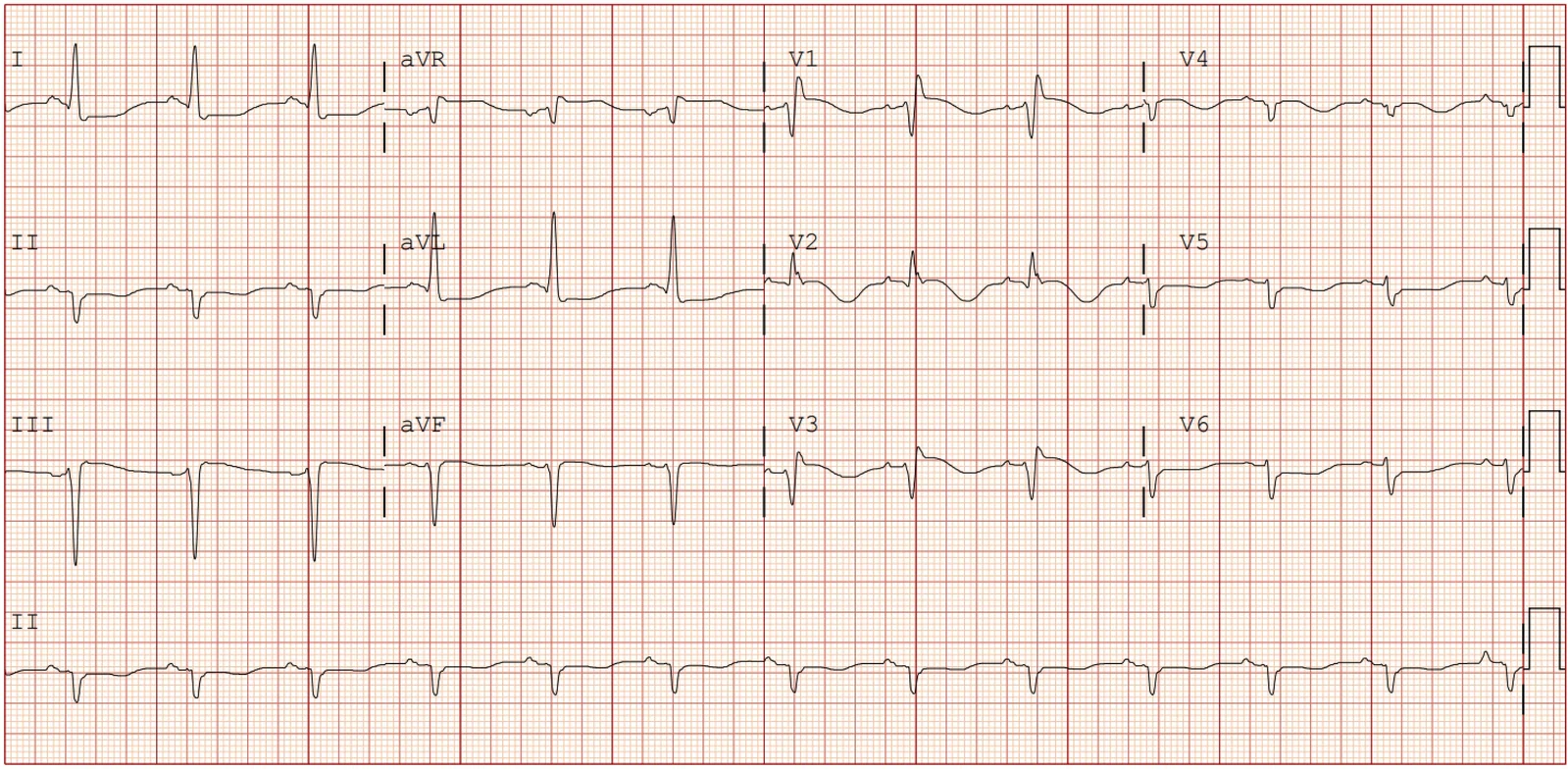
Restoration of sinus rhythm. Electrocardiography demonstrating sinus rhythm on Hospital Day 10, corresponding to 7 days after initiation of immunomodulatory therapy.

By Hospital Day 6, the patient was haemodynamically stable and no longer required vasopressor support, and the temporary transvenous pacemaker was discontinued. Because the patient presented with isolated ptosis without other focal neurological deficits, the initial clinical presentation was considered atypical for acute ischaemic stroke. Therefore, the patient was managed with close neurological observation. Due to persistent symptoms and ongoing concern for a central neurological aetiology, the Neurology team recommended brain MRI, which was subsequently obtained on Hospital Day 6. Brain MRI was subsequently obtained to evaluate for intracranial pathology and demonstrated a left thalamic acute-to-subacute lacunar infarct as well as acute-to-subacute infarcts in the left anterior cerebral artery and middle cerebral artery territories. On Hospital Day 9, transoesophageal echocardiography revealed a left atrial thrombus (representative transoesophageal echocardiogram video clip provided in Supplementary material online, *File S8*). In addition, atrial fibrillation was documented on telemetry monitoring during hospitalization, providing a potential mechanism for thrombus formation and the observed cerebral infarcts. Therapeutic anticoagulation with intravenous heparin was subsequently initiated and later transitioned to a direct oral anticoagulant prior to discharge. Despite these findings, the patient continued to demonstrate clinical and neurologic improvement. On Hospital Day 10, ECG showed restoration of sinus rhythm (*Figure 5*) with blood pressure 128/67 mmHg, heart rate 66 bpm, respiratory rate 15/min, and 100% saturation on room air.

## Discussion

Immune checkpoint inhibitor–associated myocarditis is a rare but potentially fatal irAE and is frequently accompanied by malignant arrhythmias and conduction abnormalities. While overlap syndromes involving myocarditis, myositis, and myasthenia gravis have been increasingly recognized, the occurrence of reversible complete AV block in this context remains uncommon. The present case highlights a distinctive clinical course characterized by dynamic electrocardiographic changes, including ventricular tachycardia followed by complete AV block, with subsequent recovery following prompt immunomodulatory therapy.

We hypothesize that ICI-associated myocarditis led to myocardial inflammation with subsequent troponin release and ST-segment elevation mimicking an anterior myocardial infarction. Inflammatory involvement of the cardiac conduction system likely contributed to high-grade AV block. The observed ventricular tachycardia may have reflected increased ventricular automaticity or irritability in the setting of myocardial inflammation. Suppression of ventricular arrhythmia with lidocaine may have unmasked the underlying AV conduction disturbance, resulting in the apparent emergence of complete AV block.

Alternative diagnoses, including Takotsubo cardiomyopathy, coronary vasospasm, and paraneoplastic syndromes, were considered; however, the absence of characteristic findings and the temporal association with ICI therapy supported an immune-mediated aetiology.

Although high-sensitivity troponin levels demonstrated a downward trend from Hospital Day 1, the patient experienced progressive clinical deterioration with malignant arrhythmias and conduction abnormalities. This observation underscores that troponin elevation in ICI-associated myocarditis does not necessarily correlate with disease severity or ongoing inflammatory involvement of the cardiac conduction system. The marked clinical improvement observed in this patient was temporally associated with the initiation of high-dose immunomodulatory therapy, including intravenous immunoglobulin and corticosteroids, suggesting a treatment response in immune-mediated myocardial and neuromuscular involvement.

To date, Triple-M overlap syndrome (myocarditis, myositis, and myasthenia gravis) has been reported in association with ICI therapy across a variety of malignancies. In addition, conduction abnormalities, including high-grade AV block, have been increasingly recognized as manifestations of immune checkpoint inhibitor-associated myocarditis. Ovarian cancer-associated cases appear to be uncommon in the published literature, highlighting the clinical interest of the present case.^4–10^

## Conclusion

This case illustrates a rare presentation of ICI-associated Triple-M overlap syndrome complicated by reversible complete AV block. Our findings emphasize that immune-mediated myocardial and conduction system involvement may progress despite improving cardiac biomarkers, underscoring the limitation of troponin as a sole marker of disease severity. Early recognition of overlapping immune-irAEs and prompt initiation of immunomodulatory therapy may be critical in achieving clinical recovery and reversibility of high-grade conduction abnormalities.

## Supplementary Material

ytag595_Supplementary_Data

## Data Availability

All data generated or analysed during this study are included in this published article. Additional information is available from the corresponding author upon reasonable request.

## Lead author biography

Dr. Mutsumi Kioka is a Clinical Associate Professor in the Division of Pulmonary and Critical Care Medicine at the Kirk Kerkorian School of Medicine at UNLV. He practices in the medical intensive care unit with a focus on complex cardiopulmonary conditions. His clinical and academic interests include ECG, echocardiography, and the integration of bedside imaging in critical care. He is actively involved in clinical research and medical education, with multiple peer-reviewed publications and teaching awards. He also serves as a member of the American Board of Internal Medicine Item Writing Task Force.

## Notes

**Consent:** The authors confirm that written consent for submission and publication of this case report, including images and associated text, has been obtained from the patient, in line with COPE guidance. A copy of the written consent is available for review by the Editor-in-Chief of this journal upon request.
