# Supplementary material for: Reversible complete atrioventricular block in immune checkpoint inhibitor–associated triple-M overlap syndrome: a case report
Source: Eur Heart J Case Rep. 2026 Aug 8;10(8):ytag595. doi: 10.1093/ehjcr/ytag595 (PMC13505860; doi:10.1093/ehjcr/ytag595)
Supplement: Supplementary file 1 [file ytag595_supplementary_data.zip › Supplemental File 7 Clinical Course.docx]

| **Hospital Day** | **Key Events** | **Cardiac Rhythm** | **hs-troponin I (ng/L)** | **CK (U/L)** | **Treatment** | |
| --- | --- | --- | --- | --- | --- | --- |
| **1** |  | Sinus rhythm | 22380 | 8294 |  |  |
| **2** | LHC | ST elevation | 55900 | 5442 | MP 125mg |  |
| **3** |  | VT | 32891 | 2330 | MP 1000mg | IVIG 0.4g/kg/day |
| **4** |  | Complete AVB |  | 807 | MP 1000mg | IVIG 0.4g/kg/day |
| **5** |  | V-paced | 15715 | 503 | MP 1000mg | IVIG 0.4g/kg/day |
| **6** | Pacemaker removed |  | 13412 |  | MP 500mg | IVIG 0.4g/kg/day |
| **7** | MRI Brain | Sinus rhythm |  |  | MP 250mg | IVIG 0.4g/kg/day |
| **8** |  |  | 9753 | 257 | Prednisone 40mg |  |
| **9** |  | Sinus rhythm |  |  | Prednisone 40mg |  |
| **10** |  |  | 4043 |  | Prednisone 40mg |  |
